# Distribution of Ocular Anterior and Posterior Segment Lengths Among a Cataract Surgical Population in Shanghai

**DOI:** 10.3389/fmed.2021.688805

**Published:** 2021-09-23

**Authors:** Jiao Qi, Wenwen He, Jiaqi Meng, Ling Wei, Dongjin Qian, Yi Lu, Xiangjia Zhu

**Affiliations:** ^1^Department of Ophthalmology, Eye, Ear, Nose, and Throat Hospital of Fudan University, Shanghai, China; ^2^Key Laboratory of Myopia, Ministry of Health, Shanghai, China; ^3^Shanghai Key Laboratory of Visual Impairment and Restoration, Shanghai, China; ^4^Shanghai High Myopia Study Group, Shanghai, China; ^5^Visual Rehabilitation Professional Committee, Chinese Association of Rehabilitation Medicine, Shanghai, China

**Keywords:** anterior segment length, posterior segment length, IOL Master 700, cataract patients, axial length

## Abstract

**Purpose:** To investigate the distributions of the ocular anterior and posterior segment lengths among a cataract surgical population in Shanghai.

**Design:** Cross-sectional study.

**Methods:** Ocular biometric parameters of 23,462 eyes of 23,462 cataract surgery candidates were reviewed. Axial length (AL), anterior chamber depth (ACD), and lens thickness (LT) were obtained using IOL Master. Anterior segment length (ASL = ACD + LT), posterior segment length (PSL = AL – ASL) and the ratio of ASL to PSL (ASL/PSL) were calculated.

**Results:** The mean ASL was 7.58 ± 0.39 mm, the mean PSL was 17.12 ± 2.64 mm. As the age grew, the ASL increased, and PSL increased firstly then decreased. Male subjects tended to have significantly longer ASL and shorter PSL than female subjects. With the increasing AL, the ASL was firstly decreased to trough at 20–22 mm AL group, then increased gradually, while the PSL increased rapidly. The ASL correlated positively with AL in normal, moderate and highly myopic eyes, negatively in short eyes. The PSL correlated positively with AL across the entire study population. The ASL/PSL was not constant in the eyes with different AL but had a relatively steep downward trend with the increasing AL in the short eyes, then decreased smoothly in normal, moderate and highly myopic eyes.

**Conclusions:** In Chinese cataractous eyes, longer ASL and shorter PSL were associated with elder age and male gender. The change of ASL over AL was not linear, and the ASL was smallest in the eyes with AL of 20–22 mm. The elongation of the eyeball was mainly due to the extension of the posterior segment.

## Introduction

The eye, an important visual organ, is composed of two main parts, anterior segment and posterior segment. In clinical practice, ophthalmologists noted that the ratio of the anterior segment to the posterior segment was not constant, but varied a lot in eyes with different axial length (AL). During ocular surgeries, a deep anterior chamber can be seen in small eyeballs, while a shallow anterior chamber can also be seen in eyes with long AL. This phenomenon prompts that the development of anterior and posterior segments is not synchronized. The narrow anterior segment is a relatively bigger challenge for the ophthalmologist to perform surgery on the anterior segment, and are more prone to have some intraoperative and post-operative complications. Thus, exploring the distribution of anterior and posterior segment lengths in the population can help us understand the development of anterior and posterior segments, and find out the high-risk patients and take personalized treatment.

Ocular biometric characteristics, such as AL, anterior chamber depth (ACD), white-to-white (WTW) distance, central corneal thickness (CCT), keratometric power (K), and corneal astigmatism (CA) have been described previously (1–7). However, few studies have been focused on the overall anterior and posterior segment dimensions of the eyes (8, 9). Nowadays, modern optical biometry devices can measure different axial biometric parameters of the eyes, including ACD and AL (10). IOL Master 700, based on swept-source optical coherence tomography, can provide a non-invasive image-based measurement with a 44-mm scan depth and 22-mm tissue resolution to determine the axial biometric parameters of the eye accurately (11). Consequently, the lens thickness (LT) can now be better measured with the facility, which enables the evaluation of anterior segment length (ASL = ACD + LT) and posterior segment length (PSL = AL – ASL).

Therefore, with a large number of cataract surgical candidates from Shanghai, we aim to describe the distribution of anterior and posterior segment lengths with IOL Master 700, and investigate their associations with age, sex, AL, and other ocular biometric characteristics.

## Methods

This study adhered to the tenets of the Declaration of Helsinki, and was approved by the Institutional Review Board of the Eye and Ear, Nose, and Throat (ENT) Hospital of Fudan University, Shanghai, China. Informed consents for the use of the clinical biometric data were routinely obtained from the patients before cataract surgery.

### Subjects

This study retrospectively reviewed medical records of cataract surgical candidates, aged 30 or above, at the Eye and Ear, Nose, and Throat Hospital of Fudan University from March 2018 to March 2020. The exclusion criteria were patients with previous ocular trauma or surgeries, corneal opacity, lens dislocations, other ocular diseases that could affect the measurements or those who were unable to cooperate and fixate adequately during the measurements. In this study, only one eye was counted for each patient. For patients with both eyes eligible, one eye was selected randomly. Finally, 23,462 eyes of 23,462 cataract patients were included in this analysis.

### Ocular Biometric Measurements

Ocular biometric parameters were obtained using the IOL Master 700 (Carl Zeiss AG, Jena, Germany) by experienced technicians before cataract surgery. During each measurement, the correct fixation of the examinees was visually checked on the fovea scan by the technician. The standard deviation (SD) for AL, ACD and LT were automatically calculated for each measurement, and if the SD for AL >0.027 mm, for ACD >0.021 mm or LT >0.038 mm, the device would warn of poor-quality results, which would be deleted and remeasured until reproducible readings were obtained (12).

AL was measured using signals from the tear film to the retinal pigment epithelium (RPE) of the fovea. ACD was defined as the distance from the anterior corneal surface to the anterior lens surface. LT was defined as the distance between the anterior and posterior lens surfaces. The anterior segment length (ASL) was defined as the distance from the anterior corneal surface to the posterior lens surface, calculated by the sum of LT and ACD (9, 13, 14). The distance between the posterior lens surface and the RPE of the fovea was defined as the posterior segment length (PSL), which was equal to AL minus ASL. The ratio of ASL to PSL (ASL/PSL) was also calculated.

Then, we divided all the eyes into seven groups according to the AL in this study (AL groups: <20, 20–22, 22–24.5, 24.5–26, 26–30, 30–35, ≥35 mm). The eyes with AL <22 mm were defined as short eyes, eyes with AL between 22 and 24.5 mm were defined as normal eyes, eyes with AL between 24.5 and 26 mm were defined as moderate myopic eyes, and those with AL ≥26 mm were defined as highly myopic eyes.

### Statistical Analysis

All continuous data are expressed as mean ± SD and categorical data are expressed as frequency and percentage for each category. The normality of data was tested for all ocular biometric parameters using the Kolmogorov-Smirnov (K-S) test and was considered skewed distribution if *P* < 0.05. For continuous data, differences between two groups were assessed using the Mann-Whitney *U*-test, and those among more than two groups were assessed using the Kruskal-Wallis test. For categorical data, differences were compared using the Pearson chi-squared test. The multiple linear regressions for ASL and PSL were performed, including age, sex and AL as independent variables. The correlations between anterior and posterior segment lengths with ocular biometric parameters were assessed using Pearson's correlation. *P*-values of <0.05 were considered statistically significant. All analyses and graphs were performed using IBM SPSS v22.0 (Chicago, Illinois, USA).

## Results

### Characteristics

**Figure 1:**
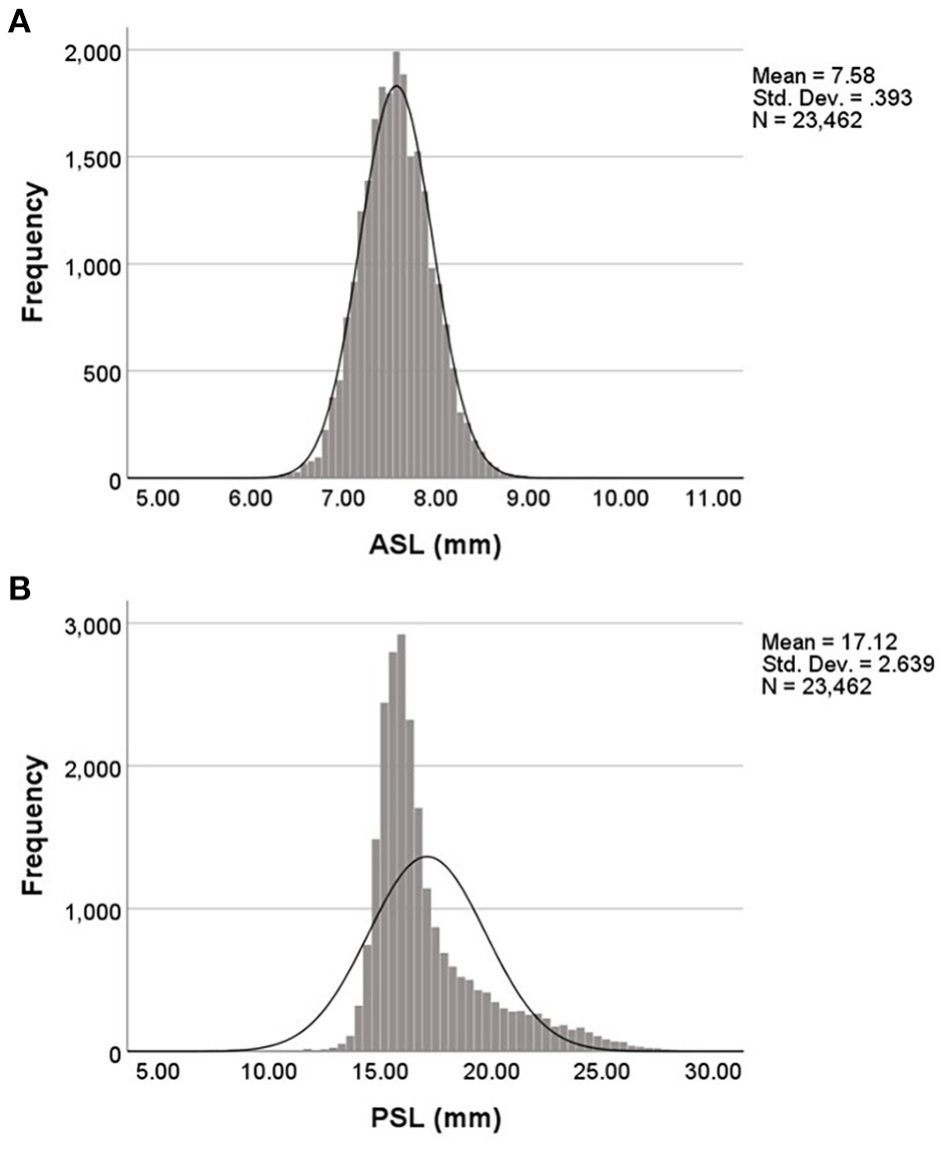
Distribution of anterior and posterior segment lengths in this study population. **(A)** The distribution of anterior segment length (ASL) was positively skewed and peaked. **(B)** The distribution of posterior segment length (PSL) was positively skewed and peaked.

Table 1 showed the general and ocular biometric characteristics of this study population. This study included 23,462 eyes (11,400 right and 12,062 left) of 23,462 cataract patients (10,210 men and 13,252 women). In this study, all the included eyes were composed of 6.0% short eyes (*n* = 1,403), 58.0% normal eyes (*n* = 13,610), 13.1% moderate myopic eyes (*n* = 3,070), and 22.9% highly myopic eyes (*n* = 5,379). The mean age was 63.5 ± 12.3, ranging from 30 to 108 years old (Supplementary Figure 1). The mean ASL in this study population was 7.58 ± 0.39 mm, the mean PSL was 17.12 ± 2.64 mm, and the mean ASL/PSL was 45.1 ± 6.2%. The distributions of ASL and PSL among this study population were shown in Figure 1, and were both skewed toward the right, especially PSL (K-S test, both *P* < 0.001).

**Table 1:** General and ocular biometric characteristics in this study population.

	**Total (*n* = 23,462)**
Age, years	63.5 ± 12.3
Sex, male/female	10,210/13,252
Eye, right/left	11,400/12,062
AL, mm	24.70 ± 2.78
CCT, μm	545 ± 36
WTW, mm	11.70 ± 0.46
ACD, mm	3.07 ± 0.48
LT, mm	4.50 ± 0.45
K, diopter	43.99 ± 1.63
CA, diopter	−1.00 ± 0.89
ASL, mm	7.58 ± 0.39
PSL, mm	17.12 ± 2.64
ASL/PSL, %	45.1 ± 6.2

*AL, axial length; CCT, central corneal thickness; WTW, white-to-white distance; ACD, anterior chamber depth; LT, lens thickness; K, keratometric power; CA, corneal astigmatism; ASL, anterior segment length; PSL, posterior segment length; ASL/PSL, the ratio of anterior segment length to posterior segment length*.

### Comparisons of Anterior and Posterior Segment Lengths Stratified by Age and Sex

**Figure 2:**
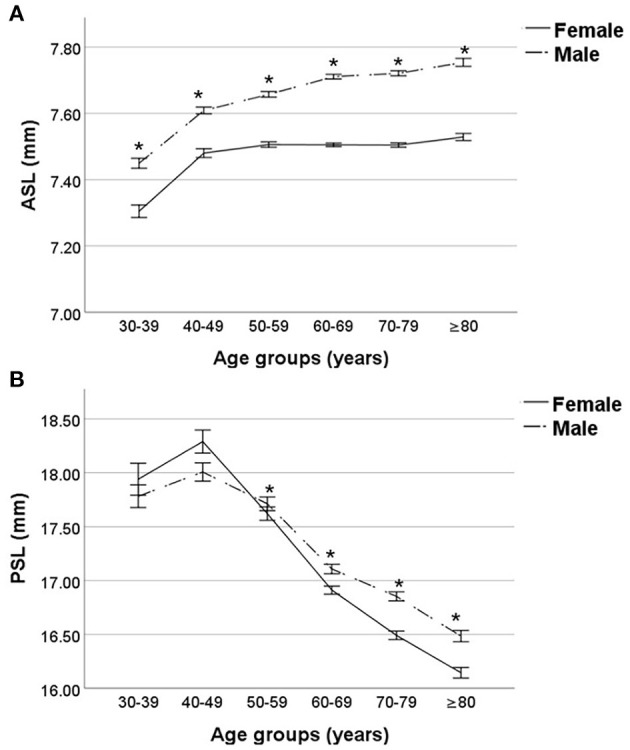
Comparisons of anterior and posterior segment lengths stratified by age and sex. **(A)** The anterior segment length (ASL) was increasing gradually both in either male or female subjects, and that of male subjects was significantly larger than that of female subjects among all age groups. **(B)** The posterior segment length (PSL) of male and female subjects both increased as the age grew from 30 to 49, then decreased with the increase of age, and male subjects had significantly longer PSL than female subjects in elder than 50 population. *Comparisons between male and female subjects, *p* < 0.05.

With the increase of age, the ASL increased both in male and female groups (Kruskal-Wallis test, both *p* < 0.001; Figure 2A). The PSL of male and female subjects both increased as the age grew from 30 to 49, then decreased with age (Kruskal-Wallis test, both *p* < 0.001; Figure 2B).

As to comparisons stratified by sex, the male subjects had significantly longer ASL than female subjects among all age groups (Mann-Whitney *U*-test, all *p* < 0.001; Figure 2A). The PSL of male subjects was significantly longer than that of female subjects in elder-than-50 population(Mann-Whitney *U*-test, all *p* < 0.001; Figure 2B). There was no significant difference in PSL between the male and female subjects in age groups 30–39 and 40–49 (Mann-Whitney *U*-test, both *p* ≥ 0.05; Figure 2B).

Multiple linear regression analysis, which included age, sex, and AL, demonstrated that larger anterior segment was associated with elder age (β = 0.006, 95% confidence interval [CI] 0.006–0.006, *p* < 0.001), male gender (β = 0.164, 95%CI 0.155–0.173, *p* < 0.001), and longer AL (β = 0.063, 95%CI 0.061–0.065, *p* < 0.001), and larger posterior segment was associated with younger age (β = −0.006, 95%CI −0.006 to −0.006, *p* < 0.001), female gender (β = −0.164, 95%CI −0.173 to −0.155, *p* < 0.001) and longer AL(β = 0.937, 95%CI 0.935–0.939, *p* < 0.001).

### Changes of Anterior and Posterior Segment Lengths With AL

**Figure 3:**
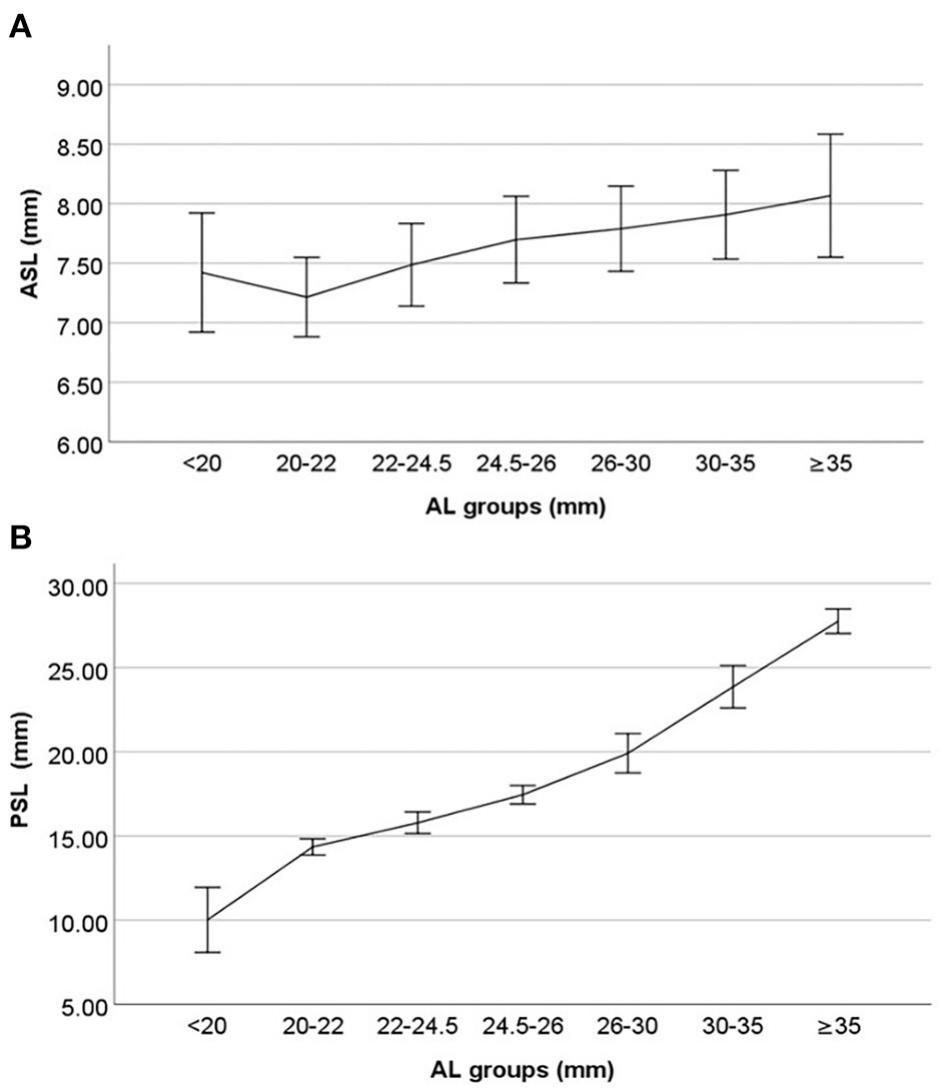
Changes of anterior and posterior segment lengths with axial length (AL). **(A)** With the increase of AL, the anterior segment length (ASL) was firstly decreased to trough in the group with AL of 20–22 mm, then increased gradually. **(B)** With the increasing AL, the posterior segment length (PSL) was always on the rise.

With the increase of AL, the ASL was firstly decreased to trough in the group with AL of 20–22 mm, then increased gradually (Kruskal-Wallis test, *p* < 0.001; Figure 3A). Meanwhile, the PSL was always on the rise as the AL increasing (Kruskal-Wallis test, *p* < 0.001; Figure 3B).

**Figure 4:**
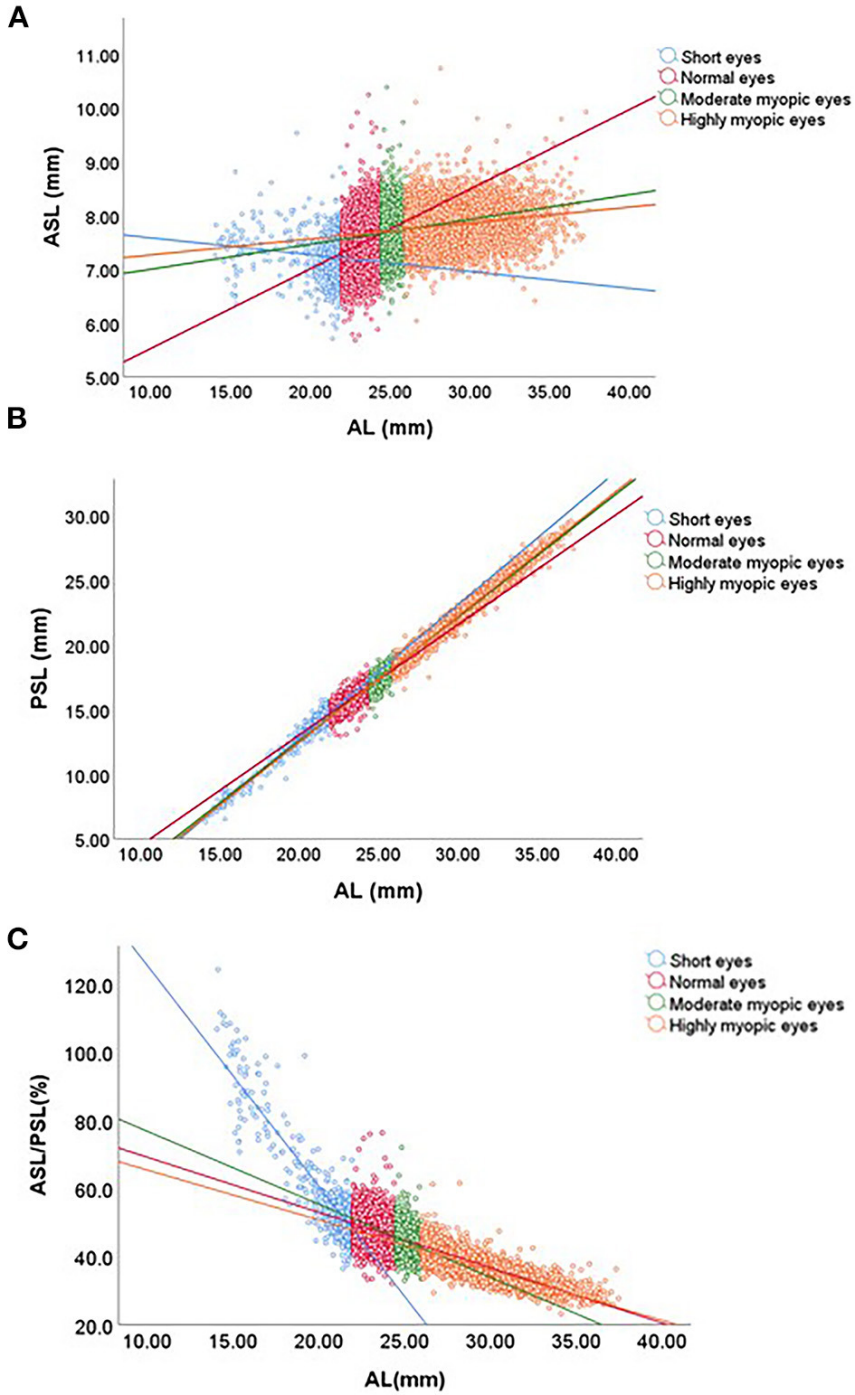
Correlations of anterior and posterior segment lengths with axial length (AL). **(A)** The anterior segment length (ASL) positively correlated with AL in the normal and myopic eyes, negatively in the short AL eyes. **(B)** The posterior segment length (PSL) positively correlated with AL in the whole population. **(C)** The ratio of ASL to PSL (ASL/PSL) had a relatively steep downward trend with the increasing AL in the short eyes, then decreased smoothly in normal, moderate and highly myopic eyes.

The Pearson's correlation analyses showed that the ASL correlated positively with AL in normal (*r* = 0.273), moderate myopic (*r* = 0.055), and highly myopic (*r* = 0.185) eyes (Pearson's correlation coefficient, all *p* < 0.05; Figure 4A). However, in the short AL group, the ASL was negatively correlated with AL (Pearson's correlation coefficient, *r* = −0.121, *p* < 0.001; Figure 4A). The PSL showed a stable increasing trend with AL across the entire study population (Pearson's correlation coefficient, all *p* < 0.001; Figure 4B).

The ASL/PSL was not constant in the eyes with different AL, but had a relatively steep downward trend with the increasing AL in the short eyes (*r* = −0.885), then decreased smoothly in normal (*r* = −0.314), moderate myopic (*r* = −0.293) and highly myopic (*r* = −0.814) eyes (Pearson's correlation coefficient, all *p* < 0.001; Figure 4C). Table 2 showed that the distribution of ASL/PSL in different AL groups. For 96.9% of eyes with AL <20 mm, the ASL/PSL was more than 50%. But the ASL/PSL was <40% for all eyes with AL >35 mm (Pearson chi-squared test, *p* < 0.001).

**Table 2:** Distribution of the ratio of anterior segment length to posterior segment length (ASL/PSL) in different axial length (AL) groups.

**AL (mm)**	**ASL/PSL (%)**		
	** <40**	**40–50**	**>50**
<20	0 (0.0%)	4 (3.1%)	127 (96.9%)
20–22	5 (0.3%)	607 (47.7%)	660 (51.9%)
22–24.5	117 (0.9%)	10,644 (78.2%)	2,849 (20.9%)
24.5–26	234 (7.6%)	2,723 (88.7%)	113 (3.7%)
26–30	2,233 (59.1%)	1,538 (40.7%)	5 (0.1%)
30–35	1,521 (99.5%)	7 (0.5%)	0 (0.0%)
≥35	75 (100.0%)	0 (0.0%)	0 (0.0%)

### Correlations of ASL and PSL With Other Ocular Biometric Parameters

Correlations of ASL and PSL with other ocular biometric characteristics were evaluated using Pearson's correlation analysis, and presented in Table 3. The results showed that the ASL correlated positively with PSL (*r* = 0.303, *p* < 0.001), CCT (*r* = 0.074, *p* < 0.001), WTW (*r* = 0.320, *p* < 0.001), ACD (*r* = 0.471, *p* < 0.001), and LT (*r* = 0.369, *p* < 0.001), negatively with the K (*r* = −0.092, *p* < 0.001), and CA (*r* = −0.024, *p* < 0.001). Meanwhile, the PSL correlated positively with ASL (*r* = 0.303, *p* < 0.001), CCT (*r* = 0.049, *p* < 0.001), WTW (*r* = 0.141, *p* < 0.001), and ACD (*r* = 0.426, *p* < 0.001), negatively with the LT (*r* = −0.187, *p* < 0.001), K (*r* = −0.230, *p* < 0.001), and CA (*r* = −0.118, *p* < 0.001).

**Table 3:** Correlations of anterior and posterior segment lengths with other ocular biometric characteristics.

	**ASL (mm)**		**PSL (mm)**	
	**β**	***P*-value**	**β**	***P*-value**
ASL, mm	–	–	0.303	0.000^*^
PSL, mm	0.303	0.000^*^	–	–
CCT, μm	0.074	0.000^*^	0.049	0.000^*^
WTW, mm	0.320	0.000^*^	0.141	0.000^*^
ACD, mm	0.471	0.000^*^	0.426	0.000^*^
LT, mm	0.369	0.000^*^	−0.187	0.000^*^
K, diopter	−0.092	0.000^*^	−0.230	0.000^*^
CA, diopter	−0.024	0.000^*^	−0.118	0.000^*^

*ASL, anterior segment length; PSL, posterior segment length; CCT, central corneal thickness; WTW, white-to-white distance; ACD, anterior chamber depth; LT, lens thickness; K, keratometric power; CA, corneal astigmatism.*

* *Statistically significant (p < 0.05)*.

## Discussion

The eyeball can be divided into the anterior segment and posterior segment. Major structures of the anterior segment include the cornea, anterior chamber, iris, and lens. Meanwhile, the posterior segment is occupied by the vitreous body, retina, choroid, and sclera. The anterior segment and posterior segment comprise tissues from different embryonic origins, for example, the lens and the cornea are derived from the surface ectoderm, but the retina is from the anterior neural plate (15). Eye development is a complex and highly regulated process. The development of the anterior segment and the posterior segment was regulated by different signaling pathways. Therefore, the anterior segment and posterior segment are not developed synchronously, so that the ratio of anterior segment to posterior segment varied a lot in the eyes with different AL. Few studies focused on the overall anterior and posterior segment dimensions of the eyes, which can help us understand the development of eyeball better. Meanwhile, narrow anterior segment is a relatively bigger challenge for the ophthalmologist to perform surgery on the anterior segment, such as phacoemulsification and filtering surgery. The patients with narrow anterior segment are more prone to some intraoperative and post-operative complications. Thus, exploring the distribution of ASL and PSL can help ophthalmologists to find out the high-risk patients and take preventions. Thus, in this study, we reviewed medical records of cataract patients elder than 30 from Shanghai, focused on the distribution of the ASL and PSL.

With the growth of age, the ASL was increased in our study population. One reasonable explanation for that might be the lens of age-related thicken. Many previous studies in western China (1), southern China (2), India (14), and Latin America (15) found that the LT increased with the growth of age. The crystalline lens is thickening due to fiber hyperplasia during the aging process (16). Meanwhile, in our study, we found that the PSL of 40–49 years old cataract patients was the longest, and the shorter PSL was more common in elder cataract patients. Some previous studies revealed that the biological characteristics of vitreous were significantly changed as the age grow (17), which may result in the shrink of the posterior segment.

In our study, we found that the anterior and posterior segment lengths had a gender bias: the male subjects were more prone to have a significantly larger anterior segment and shorter posterior segment than female subjects. The difference in ASL between males and females are similar to the differences among other ocular biometric parameters, such as AL, ACD, LT, WTW, and so on, proved in numerous previous studies of cataract population and healthy population (18–20). This phenomenon demonstrated that the anterior segment of female subjects was relatively narrow, which may explain the susceptibility to glaucoma among females (21, 22).

The ocular anterior segment is mainly occupied by the anterior chamber and crystalline lens, and the normal development of anterior segment is essential for maintaining many ocular functions, such as aqueous drainage, refraction, accommodation, and so on (23, 24). Sorbatzoglou et al. (25) reported that the mean ASL in healthy people between 31 and 44 years old was 7.36 ± 0.21 mm, and that in elder than 45 was 7.56 ± 0.24 mm. In our study, the mean ASL was 7.58 ± 0.39 mm in the cataract population, longer than healthy population, may due to the thicker lens in cataract patients. We found that compared to the PSL, the ASL in different AL eyes was relatively stable. The difference between the maximum and minimum of mean ASL among all AL groups was only 0.85 mm in this study. Small variation of the ASL regarding of the AL is essential for maintaining the normal function of the anterior segment in eyes with different AL, such as aqueous drainage. Meanwhile, the change of ASL was not linear with the AL but firstly decreased to trough in the group with AL of 20–22 mm, then increased gradually. Unexpectedly, in the short eyes, the ASL had a downward trend as the AL increasing, and the ASL of the eyes with AL between 20 and 22 mm was the smallest. It proves that as for the short eyes, the eyes with the smaller AL are more likely to be the posterior microphthalmos, which's AL is short due to reduced posterior segment dimension with normal anterior chamber dimensions (26). So that, during ocular surgeries, we often encounter that the anterior chamber of some small eyes is not narrow. This phenomenon also reminds our cataract surgeons to pay more attention to confirm whether the anterior segment is too narrow to operate when dealing with the cataract eyes with AL between 20 and 22 mm.

As for the change of PSL with AL, the PSL had a significantly increasing trend with the increasing AL in the present study. Meanwhile, we found that the ASL/PSL was not constant in the different AL eyes but had a downward trend with the increasing AL. This demonstrated that the ASL and PSL were not increasing in the same proportion, the elongation of the eyeball mainly resulted in the extension of the ocular posterior segment. Excessive lengthening of the posterior segment of the eyeball is the major structural abnormality associated with high myopia (27). Numerous previous studies about the mechanism of high myopia demonstrated that myopia-related visual signal and hypoxia signal would regulate many growth factors in the sclera, resulting in extracellular matrix remodeling of the scleral shell (28–30). That is to say, the signal that prolongs the eyeball might mainly affect the growth of the posterior segment of the eyes, but do not affect the anterior segment.

In conclusion, the present study determined the normative ocular anterior and posterior segment lengths and their correlations with sex, age, AL, and other ocular biometric characteristics in the cataract surgery candidates from Shanghai. In Chinese cataractous eyes, longer ASL, and shorter PSL were more common in elder people. Male subjects were more prone to have longer anterior segment and shorter posterior segment than female subjects. The change of ASL over AL was not linear, the ASL was smallest in the eyes with AL of 20–22 mm. The elongation of the eyeball was mainly due to the extension of the posterior segment.

## Data Availability Statement

The original contributions presented in the study are included in the article/Supplementary Material, further inquiries can be directed to the corresponding author/s.

## Ethics Statement

The studies involving human participants were reviewed and approved by the Institutional Review Board of the Eye and Ear, Nose, and Throat Hospital of Fudan University, Shanghai, China. The patients/participants provided their written informed consent to participate in this study.

## Author Contributions

XZ designed the study. JQ, WH, JM, LW, and DQ performed the study. JQ and DQ performed data collection and management. WH, JM, and LW performed data analysis and interpretation. JQ and YL wrote and reviewed the manuscript. All authors have approved the manuscript.

## Funding

This article was supported by the National Natural Science Foundation of the People's Republic of China (Grant Nos. 81870642, 81970780, 81470613, and 81670835), the Shanghai High Myopia Study Group, the Shanghai Talent Development Fund (Grant No. 201604), the Outstanding Youth Medical Talents Program of Shanghai Health and Family Planning Commission (Grant No. 2017YQ011), and the National Key R&D Program of China (Grant No. 2018YFC0116800).

## Conflict of Interest

The authors declare that the research was conducted in the absence of any commercial or financial relationships that could be construed as a potential conflict of interest.

## Publisher's Note

All claims expressed in this article are solely those of the authors and do not necessarily represent those of their affiliated organizations, or those of the publisher, the editors and the reviewers. Any product that may be evaluated in this article, or claim that may be made by its manufacturer, is not guaranteed or endorsed by the publisher.
