# Supplementary figures and images for: Distribution of Ocular Anterior and Posterior Segment Lengths Among a Cataract Surgical Population in Shanghai
Source: Front Med (Lausanne). 2021 Sep 23;8:688805. doi: 10.3389/fmed.2021.688805 (PMC8494767; doi:10.3389/fmed.2021.688805)

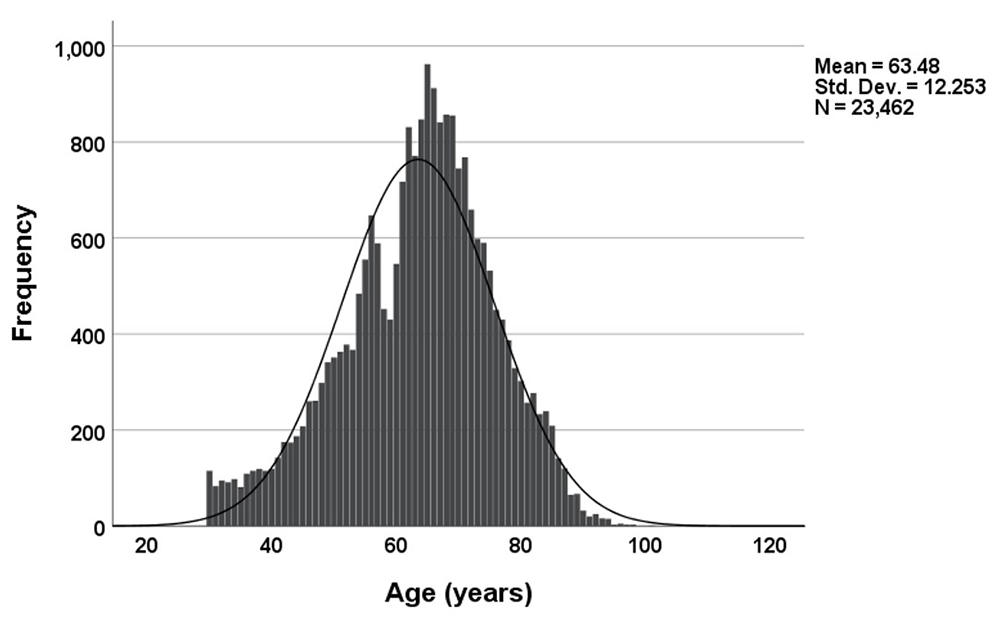

Supplement: Supplementary file 1 [file Image_1.TIF]
